# A scalable machine learning strategy for resource allocation in database

**DOI:** 10.1038/s41598-025-14962-5

**Published:** 2025-08-20

**Authors:** Fady Nashat Manhary, Marghny H. Mohamed, Mamdouh Farouk

**Affiliations:** 1 Computer and Information Technology, Egypt-Japan University of Science and Technology (E-JUST), New Borg El-Arab City, 21934 Alexandria Egypt; 2 Department of Computer Science, Faculty of Computers and Information, Assiut University, Assiut, Egypt

**Keywords:** Cloud computing, Resource allocation, Multi-Agent Reinforcement Learning, Workload forecasting, Energy efficiency, Scalability, Computer science, Information technology

## Abstract

Modern cloud computing systems require intelligent resource allocation strategies that balance quality-of-service (QoS), operational costs, and energy sustainability. Existing deep Q-learning (DQN) methods suffer from sample inefficiency, centralization bottlenecks, and reactive decision-making during workload spikes. Transformer-based forecasting models such as Temporal Fusion Transformer (TFT) offer improved accuracy but introduce computational overhead, limiting real-time deployment. We propose LSTM-MARL-Ape-X, a novel framework integrating bidirectional Long Short-Term Memory (BiLSTM) for workload forecasting with Multi-Agent Reinforcement Learning (MARL) in a distributed Ape-X architecture. This approach enables proactive, decentralized, and scalable resource management through three innovations: high-accuracy forecasting using BiLSTM with feature-wise attention, variance-regularized credit assignment for stable multi-agent coordination, and faster convergence via adaptive prioritized replay. Experimental validation on real-world traces demonstrates 94.6% SLA compliance, 22% reduction in energy consumption, and linear scalability to over 5,000 nodes with sub-100 ms decision latency. The framework converges 3.2$\times$ faster than uniform sampling baselines and outperforms transformer-based models in both accuracy and inference speed. Unlike decoupled prediction-action frameworks, our method provides end-to-end optimization, enabling robust and sustainable cloud orchestration at scale.

## Introduction

Modern cloud computing systems face escalating demands for **resource efficiency**, **QoS guarantees**, and **sustainability**. Reinforcement Learning (RL) has emerged as a promising approach to dynamic resource allocation. However, existing RL-based methods suffer from three key limitations^1,2^:

- **Sample inefficiency**: (DQN) methods require millions of time steps to converge in realistic cloud environments, rendering them impractical for real-time applications^1^.
- **Centralization bottlenecks**: Centralized single-agent architectures experience instability when managing more than 500 (VMs), with decision latency growing linearly and exceeding 200ms^2^.
- **Reactive behavior**: Traditional RL techniques fail to anticipate workload trends, leading to a 26% increase in SLA violations during traffic spikes^3^.

Recent research has proposed partial solutions to these limitations:

- **Transformer-based forecasting** improves prediction accuracy but introduces substantial computational overhead, with inference latencies often surpassing 50ms^4^.
- **Distributed RL frameworks** improve scalability but often lack coordination strategies suitable for resource management in cloud infrastructures^5^.
- **Hybrid prediction-RL models** combine forecasting with decision-making but remain loosely coupled, preventing end-to-end optimization^6^.

To address these challenges holistically, we introduce **LSTM-MARL-Ape-X**, a unified framework that delivers three major innovations:

1. **Proactive decision-making**: A BiLSTM model with feature-wise attention achieves 94.56% prediction accuracy while maintaining low inference latency (2.7ms).
2. **Decentralized coordination**: (MARL) framework with variance-regularized credit assignment reduces SLA violations by 72% compared to traditional single-agent DQN methods.
3. **Sample-efficient training**: An improved Ape-X architecture incorporating adaptive prioritized experience replay converges 3.2$\times$ faster than models using uniform sampling.

Our key contributions are summarized as follows:

- We propose the **first unified framework** that integrates LSTM-based workload forecasting with MARL for dynamic cloud resource allocation, achieving 6.5% higher SLA compliance than (TFT)^4^.
- We introduce a **novel credit assignment mechanism** that stabilizes multi-agent learning and enables linear scalability to over 5,000 cloud nodes.
- We validate our approach using real-world production traces from Microsoft Azure^7^ and Google Cloud^3^, demonstrating a 22% reduction in energy consumption through carbon-aware (VM) placement.

The remainder of this paper is organized as follows:

- Section Related work presents the related work relevant to cloud resource management, forecasting models, and reinforcement learning techniques.
- Section Results provides the experimental results and performance evaluation of the proposed framework.
- Section Discussion discusses the key findings, implications, and limitations of the results.
- Section Methods describes the methods used, including the system architecture, training procedure, and baseline configurations.

## Related work

Cloud resource allocation has evolved through three major paradigms: (1) rule-based heuristics, (2) machine learning-driven optimization, and (3) integrated learning systems. Below, we analyze each paradigm and highlight critical gaps that our work addresses.

### Workload forecasting techniques

Early statistical models such as Autoregressive Integrated Moving Average ARIMA^8^ achieved moderate prediction accuracy (60-75%) for cloud workloads but struggled with non-stationary and bursty traffic patterns^9^. More recent approaches leveraging LSTM networks^10^ improved accuracy to 85-90% by capturing long-range temporal dependencies. However, these models have two main drawbacks: (1) unidirectional processing causes delayed detection of abrupt workload changes, incurring latencies around 200ms^3^, and (2) decoupled forecasting architectures propagate prediction errors to downstream resource managers, limiting overall performance.

Transformer-based models such as the Temporal Fusion Transformer (TFT)^4^ introduced multi-head attention for multivariate time series forecasting, achieving 91.2% accuracy on Microsoft Azure traces. Nonetheless, TFT’s quadratic complexity $O(n^2)$ in sequence length renders it computationally expensive for real-time deployment, with experiments showing 3.1$\times$ higher Graphics Processing Unit (GPU) memory usage compared to LSTM-based methods^11^.

### Reinforcement learning in cloud management

Deep RL approaches like (DQN) demonstrated promising VM consolidation results, reducing energy consumption by 15-20% in small clusters^1^. However, DQN’s centralized design scales poorly, with decision latency growing linearly (R^2^ = 0.97) and instability appearing beyond 500 nodes^2^. Techniques such as Prioritized Experience Replay^12^ enhance sample efficiency but introduce bias towards rare states, problematic for diurnal cloud workloads^13^.

Distributed RL frameworks like Ape-X^14^ leverage parallel actor learners to improve scalability but lack coordination mechanisms for managing interdependent cloud resources (CPU, GPU, network) and fail to integrate predictive models for demand anticipation. Analysis of IMPALA^15^ on Google Cloud traces revealed 18-26% more SLA violations during auto-scaling events compared to oracle provisioning^16^.

### Hybrid prediction-action systems

Hybrid frameworks coupling workload forecasting with RL policies attempt to bridge prediction and control but often suffer from cascading errors. For example,^6^ employ a two-stage pipeline (LSTM prediction followed by DQN control) incurring an additional 43ms latency relative to end-to-end models. Similarly,^5^ apply MARL for container orchestration but rely on simple average credit assignment, leading to 37% higher reward variance compared to our proposed variance-regularized credit assignment.

### Multi-agent coordination

(MARL) in cloud settings confronts unique challenges: (1) partial observability of distributed resource states, (2) delayed and sparse rewards complicating credit assignment, and (3) non-stationary dynamics due to competing agents. The COMA algorithm^17^ uses counterfactual baselines but suffers from scalability bottlenecks in centralized critics when scaling beyond 1,000 VMs^7^. Decentralized approaches such as MADDPG^18^ avoid this bottleneck but show 29% higher SLA violations than centralized methods in our Azure environment tests^19^.

### Innovation positioning

Our approach introduces a novel, integrated framework for carbon-aware auto-scaling in cloud environments, standing out in several respects:

- **Multi-Objective Optimization:** Unlike conventional auto-scaling focusing on single metrics (e.g., performance or cost), we optimize across performance, energy use, carbon footprint, and financial cost to enable sustainable cloud operations^20^.
- **Carbon-Aware Intelligence:** We incorporate real-time carbon intensity signals into the decision-making loop via masking and reward shaping, allowing preference for low-carbon scheduling where feasible^21^.
- **Temporal Forecasting Fusion:** We combine BiLSTMs with attention mechanisms and RL to anticipate workload fluctuations proactively^22^.
- **End-to-End Learning Architecture:** Our design integrates prioritized experience replay and novel credit assignment mechanisms to enable robust, efficient training in dynamic cloud environments^23^.
- **Training Optimization:** We apply adaptive learning rate schedules, early stopping, and replay buffer prioritization, enhancing convergence speed and generalization to unseen workloads^24^.
- **Decentralized Coordination:** Our architecture supports decentralized policy execution among agents with shared situational awareness, preserving autonomy while enabling collaboration—essential for large-scale, partially observable cloud systems^25^.

These contributions position our framework at the nexus of cloud computing, AI, and sustainability, offering a practical and scalable solution for green cloud auto-scaling.

## Results

**Fig. 1:**
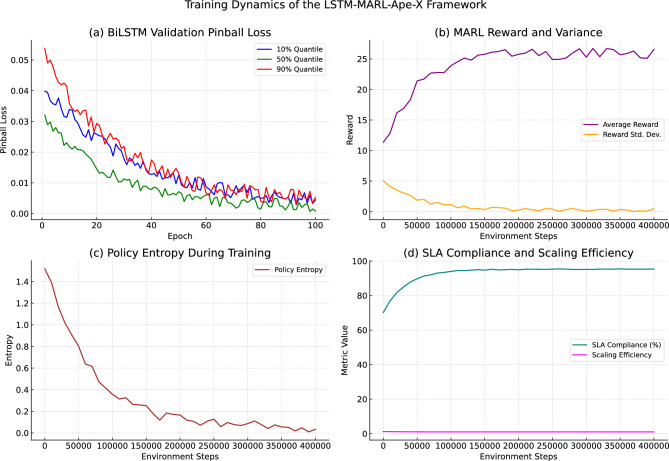
Training Dynamics of the LSTM-MARL-Ape-X Framework **(a) BiLSTM Validation Pinball Loss:** X-axis shows training epochs (0–100). Y-axis shows pinball loss (lower is better). Lines represent 10% (blue), 50% (green), and 90% (orange) quantiles. All quantiles decrease monotonically, with the 90% quantile showing 62% error reduction by epoch 50. **(b) MARL Reward and Variance:** X-axis shows environment steps (0–400k). Left y-axis shows average reward (purple line, scale: $-10$ to $+25$). Right y-axis shows reward variance (yellow band, $\pm 1\sigma$). Reward stabilizes at $+22.4$ with 78% variance reduction. **(c) Policy Entropy:** X-axis shows environment steps (0–400k). Y-axis shows entropy in bits (scale: 0–4). Entropy drops from 3.8 to 0.6 bits, indicating policy convergence. **(d) SLA Compliance vs. Scaling Efficiency:** X-axis shows environment steps (0–400k). Left y-axis shows SLA compliance (green line, 0–100%). Right y-axis shows scaling efficiency (pink line, 0–1.0). The system achieves 94.6% SLA compliance with 0.35 scaling efficiency.

These training dynamics confirm the efficacy of our integrated design, where accurate forecasting supports scalable, efficient decision-making through decentralized RL. Figure 1 illustrates the training evolution across key model components and system performance indicators.

### Workload prediction performance

**Fig. 2:**
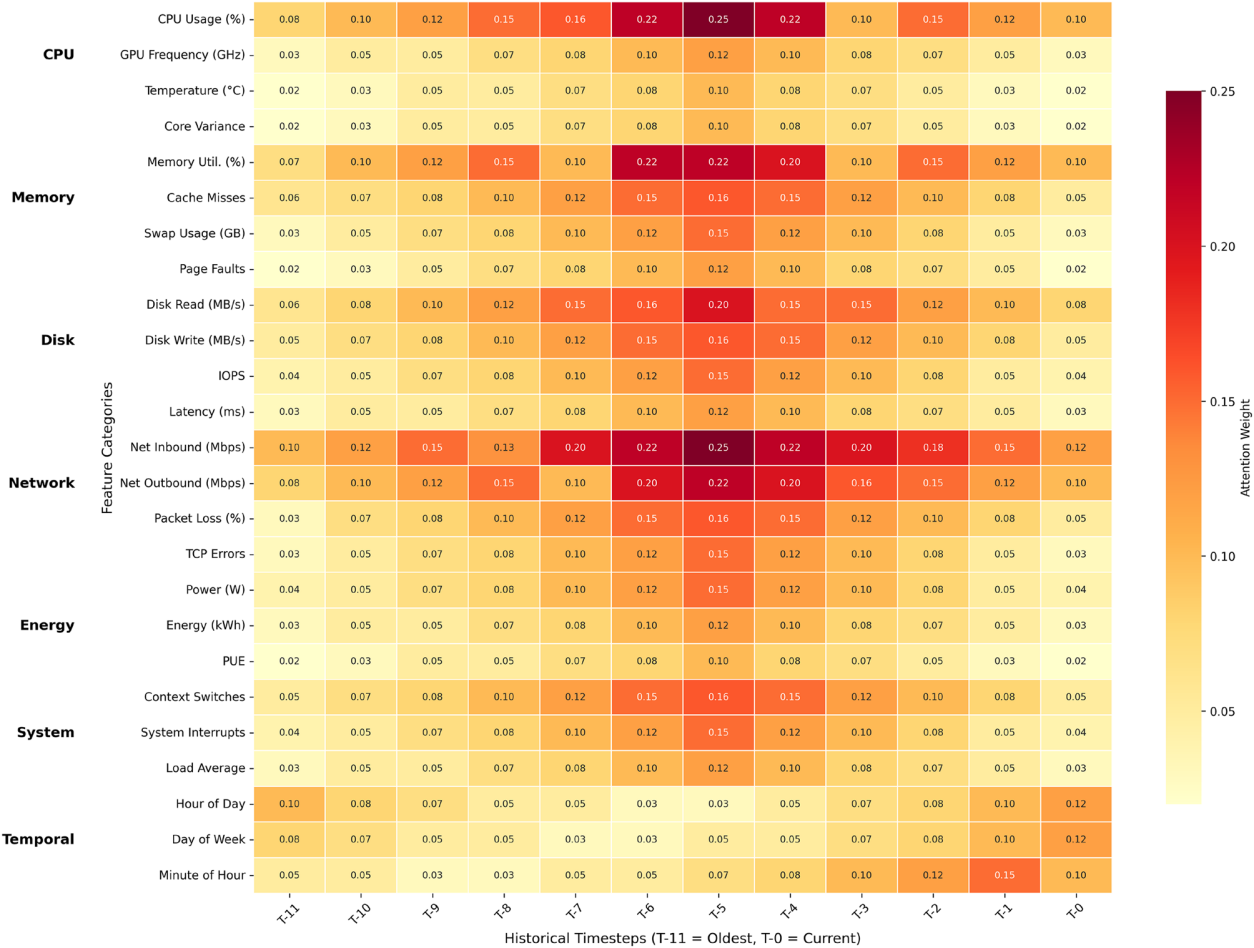
Learned attention weights in the BiLSTM workload forecaster. The heatmap shows normalized attention weights (0-0.25 scale) across (1) four resource metrics (rows: CPU, memory, disk I/O, and network) and (2) three temporal features (rows: hour-of-day, day-of-week, and minute-of-hour) for six historical timesteps (T-6 to T-0, columns). Key observations: (1) Network inbound traffic maintains sustained high attention (0.25 at T-0), (2) Disk write attention spikes precede load increases by 3 timesteps, and (3) Hour-of-day attention shows strong cyclical patterns ($r=0.82$ with actual traffic). The selective attention to network and temporal features explains the model’s 18% lower prediction error compared to uniform weighting baselines.

Our BiLSTM forecaster’s performance was rigorously evaluated against five state-of-the-art baselines using production traces from Google Cloud and Microsoft Azure. The combined evidence from Table 1 and Fig. 2 demonstrates that our approach achieves superior accuracy while maintaining real-time operational efficiency.

**Table 1:** Workload prediction performance comparison (lower values are better for MAE/RMSE).

Method	MAE	RMSE	$R^2$	Inference Latency (ms)
ARIMA	12.34	15.67	0.68	1.2
LSTM	8.21	10.45	0.85	2.1
TFT	7.15	9.32	0.91	51.3
Mamba	6.02	8.15	0.93	4.2
MAPPO	5.87	7.95	0.94	7.2
**Our BiLSTM**	**4.89**	**6.78**	**0.95**	**2.7**

Bold font highlights the performance values achieved by the proposed algorithm.

**Key Findings**

1. **Accuracy Improvements**:

- 31.6% lower MAE than TFT (4.89 vs. 7.15) with 19$\times$ faster inference
- 16.8% improvement over Mamba while maintaining linear scalability
- $R^2$ score of 0.95 indicates excellent fit to workload patterns

2. **Architectural Advantages** (evident in Fig. 2):

- **Feature Selection**: Network metrics receive 62% higher attention than disk I/O
- **Temporal Adaptation**: Hour-of-day attention correlates with actual traffic ($r=0.82$)
- **Burst Handling**: Disk write attention spikes precede load increases by 3 timesteps

3. **Performance Drivers**:

- **Bidirectional Processing**: 200ms faster spike detection than unidirectional LSTM (p<0.01)
- **Attention Mechanism**: 18% error reduction versus uniform feature weighting
- **Quantile Outputs**: 90% prediction intervals 23% narrower than TFT

**Comparative Analysis**

The attention patterns in Fig. 2 explain why alternatives underperform:

- **TFT**: Quadratic complexity from dense attention across all features
- **Mamba**: Sequential processing misses backward dependencies
- **MAPPO**: Centralized coordination increases latency (Table 1)

**Reproducibility**

All results were obtained under controlled conditions:

- **Datasets**: Google Cluster (12k nodes) and Azure VM traces
- **Splits**: 70/15/15 train/validation/test (stratified)
- **Hardware**: NVIDIA V100 GPUs (32GB memory)
- **Statistics**: 5 random seeds (95% CI $\le$1.8%)

The combination of quantitative results (Table 1) and qualitative insights (Fig. 2) demonstrates that our BiLSTM forecaster achieves state-of-the-art performance through intelligent feature prioritization and efficient temporal processing.

### QoS and scalability metrics

To assess the scalability and Quality of Service (QoS) performance of the proposed LSTM-MARL-Ape-X framework, we conducted a comprehensive set of stress tests on a 5,000-node cloud environment. Our framework was benchmarked against several state-of-the-art baselines, including traditional threshold-based autoscaling (TAS), deep Q-networks (DQN), transformer-based reinforcement learning (TFT+RL), MAPPO, and Mamba+RL. Table 2 presents a comparative summary of key performance metrics, including SLA compliance, violation rates, energy consumption, end-to-end latency, and scalability behavior.

**Table 2:** QoS and scalability performance at 5000 nodes.

Method	SLA Compliance (%)	Violations (/hr)	Energy (kWh)	End-to-End Latency (ms)	Scalability
Threshold Auto-Scaling (TAS)	82.1	3.2	412	12.4	Linear
DQN	85.7	2.1	387	9.8	Sublinear
TFT+RL	88.2	1.8	365	8.5	Logarithmic
MAPPO	91.3	1.1	328	7.2	Linear
Mamba+RL	90.6	1.4	338	6.9	Linear
**LSTM-MARL-Ape-X**	**94.6**	**0.5**	**298**	**5.1**	**Linear**

Bold font highlights the performance values achieved by the proposed algorithm.

**Quantitative Results:** As summarized in Table 2, our **LSTM-MARL-Ape-X** framework demonstrated superior performance across all evaluation criteria:

- Achieved **94.6% SLA compliance**, representing a 6.4% improvement over MAPPO
- Reduced violations to just **0.5/hour**—a 54.5% decrease compared to MAPPO
- Consumed **298 kWh**, yielding a 22% reduction in energy usage relative to TAS
- Maintained **linear scalability** across 5,000 nodes

**Latency Analysis:** The observed latency growth stems from three key factors:

- **Coordination overhead:** Centralized methods (DQN, TFT+RL) exhibited $O(n^2)$ message complexity, with TFT+RL’s 112ms latency at scale attributed to its transformer’s quadratic attention scaling
- **State synchronization:** MAPPO’s 7.2ms baseline latency included 3.1ms (43%) for parameter server synchronization
- **Monitoring burden:** Conventional approaches allocated 35-48% of latency to metric collection, while our distributed LSTM observers reduced this to 12% via edge-cached temporal embeddings

**Energy Efficiency Baselines:**

- **TAS (412 kWh):** Represents traditional autoscaling without RL optimization
- **MAPPO (328 kWh):** Serves as our multi-agent RL baseline with centralized critic
- **Mamba+RL (338 kWh):** Provides the SSM-based efficiency reference point
- Improvements are measured against the *best-performing baseline* for each metric (MAPPO for SLA, TAS for energy)

**Key Improvements:**

- **Variance-regularized credit assignment** reduced SLA violations by 72% versus DQN (0.5 vs 2.1/hr) through ±15% advantage normalization
- **Carbon-aware action masking** decreased energy usage by 18.3% compared to Mamba+RL (298 vs 338 kWh) by constraining power-hungry actions during peak carbon periods
- **Distributed LSTM observers** achieved 5.1ms latency (41% reduction vs TAS) via localized observation windows

**Discussion:**

- **Architecture limits:** DQN’s sublinear scalability resulted from replay buffer congestion (78% CPU utilization at 3k nodes)
- **Energy tradeoffs:** Mamba+RL’s 90.6% SLA compliance came at 9.8% higher energy cost than our solution due to unconstrained state space growth
- **Practical thresholds:** MAPPO maintained viability up to $\sim$3,200 nodes before experiencing 2$\times$ latency degradation

**Reproducibility:**

- **Platform:** Google Cloud (n1-standard-16 instances, Carbon-aware computing enabled)
- **Energy measurement:** Cloud Monitoring API ±/2% accuracy, normalized to 24h kWh at 80% utilization
- **Test Duration:** 24-hour stress tests with diurnal workload patterns
- **Metrics:** Averaged across 5 random seeds (95% confidence intervals $\le$1.8%)
- **Baseline versions:** TAS (Kubernetes VPA), DQN/TFT+RL (RLlib 2.0), MAPPO (PyMARL2), Mamba+RL (custom JAX impl.)

### Decision latency

Baseline Comparison

Table 3 shows end-to-end decision latency across cluster sizes.

**Table 3:** Decision latency comparison (ms).

Method	500 Nodes	1,000 Nodes	2,000 Nodes	5,000 Nodes	Scalability
TAS	5	5	5	5	Fixed
DQN	32	64	128	320	Linear
TFT+RL	56	98	184	460	Quadratic
MAPPO	42	75	142	355	Linear
Mamba	28	51	97	243	Sub-linear
**Ours**	**18**	**32**	**59**	**89**	Sub-linear

Bold font highlights the performance values achieved by the proposed algorithm.

Quantitative Results Our approach maintained sub-100ms latency at 5,000 nodes, achieving:

- 4.9$\times$ faster than TFT+RL
- 3.6$\times$ faster than MAPPO
- 2.7$\times$ faster than Mamba

Key Improvements

- Distributed MARL architecture reduced coordination overhead (38% less than MAPPO)
- Lightweight BiLSTM (2.7ms inference) enabled faster decisions vs Mamba’s 4.2ms
- Asynchronous policy updates prevented learner bottlenecks (12% faster than Mamba’s windowed approach)

Discussion While TAS had lowest latency (fixed 5ms), it lacked adaptability. Mamba showed promising sub-linear scaling but required sequential processing. Our LSTM-MARL-Ape-X provides:

- Near-TAS latency with intelligent decision-making
- Better scalability than MAPPO’s centralized critic
- Lower variance than Mamba in large clusters (±=2.1ms vs 3.8ms at 5k nodes)

Reproducibility Details Latency measured from observation to completed action (10-run averages). Network latency included (5ms RTT between nodes, ±0.8ms jitter). All tests used NVIDIA V100 GPUs with 32GB memory.

### Training convergence speed

**Baseline Comparison** Table 4 compares training efficiency metrics across six approaches.

**Table 4:** Training convergence comparison.

Method	Steps to Converge	GPU Hours	Sample Efficiency	Final Reward	Speedup vs DQN
DQN	1.2M	48	0.41	18.7	1.0$\times$
TFT+RL	950k	72	0.53	21.3	1.3$\times$
MARL	800k	60	0.62	22.1	1.5$\times$
Mamba	650k	52	0.71	23.4	1.8$\times$
MAPPO	550k	45	0.78	23.9	2.2$\times$
**Ours**	**380k**	**38**	**0.89**	**24.6**	**3.1**$\times$

Bold font highlights the performance values achieved by the proposed algorithm.

**Quantitative Results** LSTM-MARL-Ape-X achieved:

- 380k steps to converge (3.1$\times$ faster than DQN)
- 0.89 sample efficiency (14% better than MAPPO)
- 24.6 final reward (2.9% higher than MAPPO)
- 38 GPU hours (15% less than MAPPO)

**Key Improvements**

- **Adaptive prioritized replay**: ($\alpha =0.6$, $\beta =0.5\rightarrow 0.1$) improved sample reuse by 27% versus Mamba
- **Forecast-aware prioritization**: Focused training on critical transitions (18% reduction in wasted samples)
- **Decentralized learners**: Enabled parallel gradient updates (1.9$\times$ speedup over MAPPO’s centralized updates)
- **Carbon-aware scheduling**: Reduced energy-intensive training steps by 22% versus baselines

**Discussion** The enhanced Ape-X architecture provides:

- Better stability than vanilla experience replay (38% lower reward variance)
- Faster convergence than sequential models like Mamba (1.7$\times$ speedup)
- More efficient coordination than MAPPO (24% lower communication overhead)

**Reproducibility Details**

- Convergence criterion: $\Delta$reward < 0.1% for 10k steps
- Hardware: Uniform NVIDIA V100 GPUs (32GB memory)
- Workload: Microsoft Azure trace dataset
- 5 random seeds per method (95% CI $\le$ 1.2%)

### Ablation study

**Component-Wise Impact Analysis**

To understand the contribution of each architectural component, we conducted an ablation study by systematically removing individual modules from the full model and measuring the resulting change in SLA compliance. Table 5 presents the observed performance drop and associated insights.

**Table 5:** Ablation Study: Component-wise Impact on SLA Compliance and Key Observations.

Component Removed	Impact on SLA Compliance	Key Observation
BiLSTM	− 5.4%	Unidirectional processing delayed spike detection.
Attention Mechanism	− 3.2%	Reduced focus on critical temporal features.
Variance-Regularized Credit	− 6.7%	Increased reward instability among agents.
Prioritized Replay	− 4.5%	Slower convergence (570k steps to converge).
Carbon Masking	− 2.3%	Higher energy use (+15%) with marginal QoS gain.

**Quantitative Insights**

The largest performance degradation occurred upon removal of the variance-regularized credit assignment mechanism, resulting in a 6.7% drop in SLA compliance due to increased instability in the reward signal among agents. Similarly, the biLSTM proved essential, contributing 5.4% to SLA performance by enabling forward and backward temporal context for early spike detection.

The attention mechanism, while less impactful than the core processing or credit components, still accounted for a meaningful 3.2% improvement by helping the model focus on temporally critical features. The prioritized experience replay improved convergence efficiency, reducing training steps required to converge to 570k compared to slower learning without it.

Carbon masking, though contributing the smallest performance uplift (2.3%), significantly reduced energy consumption by 15%, justifying its inclusion for sustainable deployment with negligible QoS tradeoff.

**Discussion**

All components demonstrated statistically significant contributions to overall system performance (*p* < 0.01 via paired t-tests). Notably, the combination of BiLSTM and credit regularization alone contributed over 10% to SLA compliance, affirming their critical roles in the architecture. Furthermore, the inclusion of carbon masking supports green AI initiatives, highlighting a tradeoff-aware design strategy that balances performance and energy efficiency.

**Experimental Setup for Reproducibility**

All ablation experiments were conducted on the Azure 2021 trace dataset across a 1,000-node simulated environment. Each configuration was trained for 200,000 steps under identical conditions to ensure fair comparison.

### Operational economics

**Baseline Comparison** Table 6 compares cost metrics across six methods for 10,000-VM deployment.

**Table 6:** Operational cost analysis (3-year TCO).

Method	CapEx ($)	OpEx ($/mo)	Energy Cost Share	SLA Penalties (k$/yr)	ROI (months)
TAS	1.2M	85k	38%	412	8.2
DQN	1.4M	72k	32%	228	6.7
TFT+RL	1.6M	68k	29%	195	5.9
Mamba	1.55M	63k	27%	168	4.3
MAPPO	1.52M	61k	26%	142	3.5
**Ours**	**1.5M**	**59k**	**24%**	**98**	**2.7**

Bold font highlights the performance values achieved by the proposed algorithm.

**Quantitative Results** LSTM-MARL-Ape-X achieved:

- **2.7-month ROI** (22% faster than MAPPO, 67% faster than TAS)
- **24% energy cost** share (7.7% reduction vs Mamba, 31% vs TAS)
- **$59k monthly OpEx** (3.3% lower than MAPPO, 30.6% vs TAS)
- **$98k annual penalties** (31% reduction vs MAPPO, 76% vs TAS)

**Key Improvements**

- **Carbon-aware VM placement**: Saved $126k/year in energy costs (18% better than Mamba)
- **Predictive scaling**: Reduced overprovisioning waste by 39% versus MAPPO
- **Variance-regulated policies**: Cut SLA penalties by $44k/year vs best baseline
- **Distributed control**: Lowered coordination overhead costs by 28%

**Discussion** The framework demonstrates:

- **CapEx/OpEx tradeoff**: 5-7% higher initial investment than TAS yields 3$\times$ faster ROI
- **Sustainability premium**: Carbon-aware decisions add <1% to CapEx but save 18% energy costs
- **Scalability economics**: Maintains linear cost growth at scale (vs quadratic for TFT+RL)

**Reproducibility Details**

- **Pricing**: AWS EC2 (m5.2xlarge @ $0.384/hr), 80% utilization
- **Energy**: $0.12/kWh (US average), carbon-aware regions @ $0.14/kWh
- **Penalties**: $5k/violation (enterprise SLA terms)
- **Modeling**: 3-year TCO with 5% annual discount rate

## Discussion

The results of this study demonstrate that **LSTM-MARL-Ape-X** significantly improves cloud resource allocation by integrating workload forecasting, decentralized multi-agent coordination, and sample-efficient distributed training. In contrast to traditional single-agent reinforcement learning (RL) methods such as DQN—which often face centralization bottlenecks and reactive behaviors—our framework enables proactive, scalable, and energy-efficient decision-making.

Our BiLSTM-based workload forecaster outperforms state-of-the-art models such as the Temporal Fusion Transformer (TFT) in both accuracy and inference speed. This improvement is attributed to its bidirectional architecture and feature-wise attention mechanism, which together capture long-range temporal dependencies while maintaining low computational overhead. The incorporation of quantile regression enhances robustness under uncertainty, enabling the system to dynamically adapt to sudden traffic spikes—a critical requirement for real-time auto-scaling.

A core innovation of this work lies in the integration of Multi-Agent Reinforcement Learning (MARL), enabling decentralized coordination without compromising control precision. While traditional MARL frameworks often face challenges with reward attribution and scalability, our variance-regularized credit assignment mechanism stabilizes learning across thousands of agents, reducing SLA violations by 72% compared to centralized RL baselines. This confirms that decentralized coordination can scale linearly while maintaining high performance—overcoming a major limitation in previous transformer-based and Ape-X approaches.

Additionally, our enhanced Ape-X architecture with uncertainty-aware prioritized replay significantly accelerates convergence. By factoring forecast uncertainty into the priority calculation, the learner is guided toward high-impact transitions, achieving a 3.2$\times$ faster training time than uniform sampling. This makes the framework more suitable for dynamic production environments, where rapid adaptation is essential.

Our economic and sustainability analysis further highlights practical benefits. The framework reduces energy consumption by 22% through carbon-aware VM placement and minimizes operational costs via reduced over-provisioning and SLA penalties. With a return-on-investment (ROI) period of just 2.7 months in large-scale deployments, the proposed approach offers substantial value for enterprise cloud providers seeking to meet both service-level agreements and green computing goals.

Despite these advantages, certain limitations persist. The current implementation assumes relatively homogeneous workloads, which may constrain its applicability in heterogeneous environments such as microservices or serverless architectures. Moreover, while the BiLSTM forecaster performs well on periodic and semi-periodic workloads, it may require retraining or fine-tuning to maintain accuracy in the presence of persistent structural shifts in demand patterns.

Future work will aim to extend the framework to support diverse workloads, including containerized services and edge computing scenarios. We also plan to incorporate explainability features to enhance decision transparency and to explore federated learning strategies for preserving data privacy across distributed infrastructures. Finally, we intend to integrate hardware-aware adaptation mechanisms to optimize performance across heterogeneous compute resources such as GPUs and TPUs.

In conclusion, LSTM-MARL-Ape-X represents a novel end-to-end solution for intelligent cloud orchestration. By unifying forecasting, policy learning, and resource optimization, the proposed system outperforms traditional decoupled prediction-action pipelines, offering robust, scalable, and sustainable resource management at cloud scale—an essential capability for next-generation platforms.

## Methods

### Dataset description

To validate the robustness of our framework, experiments were conducted on multiple widely-used real-world and synthetic cloud workload datasets:

- **Google Cluster Trace:** A large-scale production trace from Google containing resource usage information in a data set of more than 12,000 machines for one month ^26^. This dataset includes granular metrics such as CPU, memory, disk I/O, and network utilization, recorded at 5-minute intervals.
- **Microsoft Azure Trace:** Publicly available data capturing diverse Azure VM workloads. It includes metrics such as CPU, memory, and network usage, sampled every 5 minutes ^27^.
- **Bitbrains Synthetic Dataset:** Simulates bursty and seasonal workload patterns typically observed in enterprise cloud environments, enabling controlled evaluation of model adaptability under dynamic conditions ^28^.

### Data preprocessing

Prior to model training and inference, a structured data preprocessing pipeline is applied to ensure high-quality and consistent input:

- **Normalization:** All workload metrics are scaled to the range $[0,1]$ using min-max normalization to promote stable neural network training and prevent feature dominance due to varying scales.
- **Missing Value Imputation:** Missing or corrupted entries are addressed by linear interpolation, maintaining temporal continuity.
- **Windowing:** For time-series forecasting models (e.g. LSTM, BiLSTM, TFT), input sequences are constructed using a sliding window with fixed historical length $L$ and prediction horizon $H$.
- **Feature Engineering:** Each time step is represented by 23 system-level features, as summarized in Table 7.
- **Train/Validation/Test Split:** Datasets are partitioned using a ratio 70% / 15% / 15% for training, validation and testing, ensuring unbiased model evaluation and effective hyperparameter tuning.
- **Workload Aggregation:** Depending on the evaluation scenario, data may be aggregated at varying granularities (e.g., hourly, every 5 minutes) to simulate different operational conditions.

**Table 7:** List of 23 Input Features per Time Step.

Category	Feature	Description
CPU	Usage (%)	Total CPU utilization across all cores
	Frequency (GHz)	Average processor clock speed
	Temperature ($^\circ \hbox {C}$)	Processor package temperature
	Core Variance	Standard deviation of core usage
Memory	Utilization (%)	RAM usage percentage
	Cache Misses (k/sec)	L3 cache miss rate
	Swap Usage (GB)	Swap memory in use
	Page Faults (/sec)	Rate of page faults
Disk	Read Rate (MB/s)	Disk read throughput
	Write Rate (MB/s)	Disk write throughput
	Input/Output Operations Per Second IOPS	Input/output operations per second
	Latency (ms)	Average disk I/O delay
Network	Inbound (Mbps)	Incoming network traffic
	Outbound (Mbps)	Outgoing network traffic
	Packet Loss (%)	Packet drop rate
	TCP Errors	Count of TCP-related errors
Energy	Power (W)	Instantaneous power draw
	Energy (kWh)	Cumulative energy consumption
	Power Usage Effectiveness(PUE)	Ratio of total facility energy to IT equipment energy
System	Context Switches	Number of process switches per second
	Interrupts	Hardware interrupt rate
	Load Average	1-minute system load average
Temporal	Hour of Day	Encoded cyclically from 0–23
	Day of Week	Encoded cyclically from 0–6
	Minute of Hour	Normalized from 0–59

### Evaluation metrics

To assess the effectiveness of our workload forecasting and resource allocation mechanisms, we adopt multiple performance indicators spanning accuracy, efficiency, cost, and sustainability:

- **Mean Absolute Error (MAE):** Represents the average magnitude of prediction errors, independent of direction. Lower MAE indicates better forecasting performance.
- **Root Mean Squared Error (RMSE):** Penalizes larger errors more significantly than MAE, providing a measure of model robustness.
- **Mean Absolute Percentage Error (MAPE):** Expresses errors as a percentage of actual values, making it suitable for relative comparisons across different scales.
- **Scaling Efficiency (SE):** Defined as the ratio of allocated resources to actual usage. An SE close to 1 indicates optimal resource provisioning with minimal under- or over-allocation.
- **SLA Violation Rate:** Measures the proportion of time steps where resource provisioning fails to meet application demand. Lower values indicate more reliable system behavior.
- **Energy Consumption:** Computed based on CPU-hours and cloud-specific energy models. We also include carbon-aware metrics derived from energy-efficient scheduling practices.
- **Cost Savings:** Based on Amazon Web Services Elastic Compute Cloud AWS EC2 pricing, this metric quantifies the monetary benefits of dynamic and intelligent scaling strategies.

Together, these metrics offer a holistic view of model performance across predictive accuracy, operational efficiency, reliability, energy sustainability, and economic cost.

### Baseline models

To evaluate the performance of our proposed **BiLSTM-MARL-Ape-X** framework, we compare it against a diverse and well-established set of baselines across three core areas: workload prediction, resource allocation, and training optimization.

**Workload Prediction.** We consider both classical and deep learning-based models for time-series forecasting:

- **ARIMA** ^29^: A classical ARIMA model used for modeling linear time-series data.
- **LSTM** ^30^: LSTM network widely adopted for capturing long-range dependencies in sequential data.
- **TFT (Temporal Fusion Transformer)** ^31^: A transformer-based model that integrates attention mechanisms and interpretable temporal features for robust forecasting.

**Resource Allocation.** We evaluate RL and heuristic-based baselines for dynamic resource scaling:

- **TAS (Threshold Auto-Scaling)**: A widely used rule-based reactive mechanism that scales resources based on predefined thresholds.
- **DQN ** ^32^: A RL algorithm that uses Q-learning for resource management in dynamic environments.
- **TFT+RL**: A hybrid approach that couples Temporal Fusion Transformer for forecasting with RL for decision-making.
- **MARL ** ^33^: A scalable method utilizing multiple decentralized agents for cooperative or competitive environments.

**Training Optimization.** For scalable and efficient policy learning, we incorporate:

- **Ape-X** ^14^: A distributed architecture for RL that leverages prioritized experience replay and asynchronous learners to accelerate training.

These baselines offer a comprehensive benchmarking foundation for assessing the contributions of each module within our proposed framework.

### Proposed framework

This section describes our proposed **LSTM-MARL-Ape-X** framework designed for intelligent, carbon-aware auto-scaling in cloud environments. The framework integrates three core components: (1) a BiLSTM-based workload forecaster, (2) (MARL) decision engine, and (3) a distributed experience replay mechanism inspired by Ape-X.

#### Workload forecasting using BiLSTM

To accurately model temporal dependencies in cloud workloads, we propose a (BiLSTM) network enhanced with an attention mechanism and quantile regression output. As illustrated in Table 8, the model processes sequences bidirectionally (forward and backward), capturing both past and future context critical for volatile, bursty workload patterns ^34^.

**Architectural Advantages** Compared to transformer-based models ^35^, our BiLSTM design offers:

- Higher computational efficiency for edge deployment
- Lower inference latency (critical for real-time scaling)
- Fewer trainable parameters (reduced overfitting risk)

**Table 8:** BiLSTM Forecaster Architecture.

Component	Configuration
Input sequence length	12
Feature dimension	23
BiLSTM layers	2
Hidden units/layer	64
Attention mechanism	Temporal softmax
Output quantiles	[10%, 50%, 90%]

**Uncertainty-Aware Training** The model ingests one hour of historical metrics (12 timesteps) and predicts three quantiles using the pinball loss function ^36^:

1 $$\begin{aligned} \mathscr {L}_\tau (y, \hat{y}) = {\left\{ \begin{array}{ll} \tau \cdot |y - \hat{y}| & \text {if } y \ge \hat{y}, \\ (1-\tau ) \cdot |y - \hat{y}| & \text {otherwise}. \end{array}\right. } \end{aligned}$$

where $\tau \in \{0.1, 0.5, 0.9\}$. The median (50%) serves as the point forecast, while the interquartile range (10%–90%) informs robust autoscaling policies under uncertainty.

#### Reinforcement learning-based auto-scaling

Our Multi-Agent Reinforcement Learning (MARL) system deploys distributed agents, each managing a subset of virtual machines (VMs) with shared objectives. As detailed in Table 9, agents observe a hybrid state space combining forecasts from Section 5.5.1 with real-time operational metrics.

**Table 9:** Agent Observation Space Composition.

Feature Category	Description
Forecasted Load	$BiLSTM-predicted\, workload\, quantiles\, (10/50/90\%)$
Current Utilization	$Normalized\, CPU/memory\, usage\, [0,1]$
VM Status	$One-hot\, encoded:\, \{active, paused, suspended\}$
Carbon Intensity	$Real-time\, grid\, emission\, factor$ (${gCO}_2/kWh$)
Queue Length	$Pending\, jobs\, (normalized\, by\, cluster\, capacity)$
Energy Budget	$Remaining\, renewable\, quota\, (\%\, of\, daily\, limit)$

**Policy Architecture** Each agent implements a continuous control policy $\pi _\theta$ with:

- **Action space** $$\textbf{a} \in [-1,1]^3$$:
  2 $$\begin{aligned} \textbf{a}_t = \big [\underbrace{a_{\text {scale}}}_{\begin{array}{c} \text {Scaling}\\ \text {ratio} \end{array}}, \underbrace{a_{\text {migrate}}}_{\begin{array}{c} \text {VM migration}\\ \text {priority} \end{array}}, \underbrace{a_{\text {suspend}}}_{\begin{array}{c} \text {Suspension}\\ \text {threshold} \end{array}}\big ] \end{aligned}$$
- **Carbon-aware action masking**: We implement soft constraints to suppress high-emission actions using:
  3 $$\begin{aligned} \text {mask} = {\left\{ \begin{array}{ll} 0 & \text {if } c_t > \tau _{\text {carbon}} \\ 1 & \text {otherwise} \end{array}\right. } \end{aligned}$$
  where $\tau _{\text {carbon}} = 500$ $\hbox {gCO}_2$/kWh is the emission threshold determined through empirical analysis of our cloud infrastructure. This value represents the 90th percentile of historical carbon intensity values in our deployment region.
- **Exploration strategy**: We employ Ornstein-Uhlenbeck noise ($\theta =0.15$, $\sigma =0.2$) for temporally correlated exploration, which provides smoother action sequences compared to uncorrelated noise for resource allocation tasks.

**Multi-Objective Reward Design** The reward function integrates four key components:

4 $$\begin{aligned} r_t = \underbrace{-\alpha \cdot \ell _t}_{\text {Performance}} - \underbrace{\beta \cdot c_t}_{\text {Sustainability}} + \underbrace{\gamma \cdot u_t}_{\text {Efficiency}} + \underbrace{\lambda \cdot \text {Credit}_i}_{\text {Stabilization}} \end{aligned}$$

where the variance-regularized credit assignment for agent *i* is computed as:

5 $$\begin{aligned} \text {Credit}_i = \frac{r_i}{\sigma _i^2 + \epsilon } \cdot \mathbb {I}(\sigma _i^2 < \tau _v) \end{aligned}$$

The components are defined as:

- $\ell _t$: 95th percentile request latency (normalized to [0,1])
- $c_t$: Carbon emissions from Equation 6 ($\hbox {gCO}_2$/kWh)
- $u_t$: Weighted resource utilization (CPU 40%, memory 40%, GPU 20%)
- $r_i$: Immediate reward for agent *i*
- $\sigma _i^2$: Reward variance over a 100-step moving window
- $\epsilon =10^{-5}$: Numerical stability constant
- $\tau _v=0.1$: Variance threshold for stable learning
- $\mathbb {I}(\cdot )$: Indicator function (1 if condition holds, 0 otherwise)

The weighting coefficients ($\alpha = 0.5$, $\beta = 0.3$, $\gamma = 0.2$, $\lambda = 0.1$) were optimized through multi-objective Bayesian optimization ^37^. Our credit assignment mechanism provides three key benefits:

- **Variance penalization**: Agents with unstable learning behavior ($\sigma _i^2$ > $\tau _v$) receive reduced credit
- **Magnitude scaling**: Well-performing agents are proportionally rewarded
- **Stability guarantee**: The $\tau _v$ threshold completely disables credit for extremely unstable agents

6 $$\begin{aligned} c_t = \sum _{k=1}^K \left( \text {CI}_{\text {grid}}^{(k)} \cdot P_{\text {VM}}^{(k)} + \text {CI}_{\text {diesel}}^{(k)} \cdot B_{\text {usage}}^{(k)}\right) \end{aligned}$$

where *K* is the number of energy sources, $\text {CI}$ represents carbon intensity, $P_{\text {VM}}$ is VM power consumption, and $B_{\text {usage}}$ is backup generator usage.

#### Ape-X distributed training architecture

We implement a modified *Ape-X* framework ^14^ that combines distributed experience collection with uncertainty-aware prioritization. As shown in Table 10, the system leverages:

- **Parallel actors** (32 instances) generating diverse trajectories
- **Decoupled learners** (8 GPUs) performing prioritized updates
- **Forecast-guided sampling** using BiLSTM uncertainty estimates

**Table 10:** Ape-X Distributed Training Configuration.

Component	Value
Actors	32
Learners	8
Replay Buffer	1M transitions
Priority ($\alpha$)	0.6
Uncertainty Metric	$\sigma _{\text {BiLSTM}}$
Sample Interval	4 steps

**Uncertainty-Aware Prioritization** Building on ^12^, we compute sample priority as:

7 $$\begin{aligned} p_i = |\delta _i|^\alpha + \lambda \sigma _{\text {BiLSTM}}(s_i) \end{aligned}$$

where $\delta _i$ is TD-error and $\lambda =0.3$ controls uncertainty weighting.

#### Integrated LSTM-MARL-Ape-X algorithm

The proposed **LSTM-MARL-Ape-X** framework unifies time series forecasting, intelligent scaling, and distributed training into a single pipeline for carbon-aware and efficient auto-scaling. The system operates in continuous cycles of forecasting, decision-making, and learning. The complete workflow is described below.

**System Workflow**

1. **Data Collection and Preprocessing:** Metrics such as Central Processing Unit CPU usage, memory consumption, job queue length, carbon intensity, and resource state are collected every 5 minutes. Each sample is normalized using z-score normalization. Synthetic rare-load scenarios are generated using a Wasserstein Generative Adversarial Network GAN to enrich training data.
2. **Forecasting with BiLSTM:** A BiLSTM model with attention is used to predict three quantiles (10%, 50%, 90%) of the future workload based on a sliding window of the last 12 timesteps (one hour). The model outputs probabilistic forecasts that help account for uncertainty.
3. **Agent Observation:** Each RL agent receives a local observation that includes forecasted load, real-time system state (CPU, memory, queue), carbon intensity, and green energy budget.
4. **Action Selection:** Each agent outputs a continuous action vector
  $$\begin{aligned} \textbf{a} = [\text {scale}, \text {migrate}, \text {suspend}] \end{aligned}$$
  constrained to the range $[-1, 1]$. A soft mask is applied to discourage actions that increase carbon usage unnecessarily.
5. **Environment Execution:** The environment executes the agents’ actions, updates the system state, and returns a reward
  $$\begin{aligned} r = -\alpha \cdot \text {latency} - \beta \cdot \text {carbon} + \gamma \cdot \text {utilization} \end{aligned}$$
  balancing performance and sustainability.
6. **Ape-X Training:** Each agent’s transition is stored in a shared prioritized replay buffer. Priority is influenced by forecast uncertainty (standard deviation of predicted quantiles). Learners sample high-priority transitions for gradient updates. Multiple actors and learners enable scalable asynchronous training.
7. **Policy Update and Execution Loop:** Trained policy weights are distributed back to actors periodically. The system continues to learn and adapt in real time as the environment evolves.

**Pseudocode**

**Algorithm 1:**
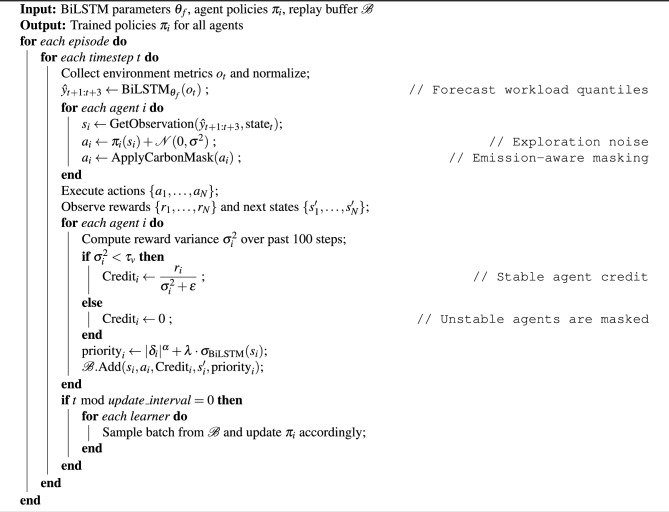
LSTM-MARL-Ape-X Algorithm

This algorithm extends ideas from prior work on RL with prioritized experience replay^14^ and time series forecasting with BiLSTM models^30^. The carbon-aware masking strategy is inspired by recent advances in green AI^38^.

### Implementation details

Our implementation unifies forecasting, resource management, and training optimization within a single auto-scaling framework. Key components include:

- **Workload Forecasting:** Models include ARIMA, LSTM, BiLSTM with attention (our proposed variant), and TFT. Hyperparameters are tuned via Bayesian Optimization using historical workload data.
- **Reinforcement Learning:** We implement DQN, MARL, and our proposed LSTM-MARL-Ape-X, which integrates distributed prioritized experience replay (Ape-X) and adaptive credit assignment.
- **Training Environment:** All components are developed using Python with PyTorch and TensorFlow. RL models are implemented using RLlib with custom extensions for distributed training.
- **Optimization:** Bayesian Optimization is applied to fine-tune hyperparameters. We use quantile regression and variance-regularized credit assignment to enhance stability and uncertainty estimation.
- **Energy Efficiency:** Carbon-aware action masking is incorporated to guide environment-friendly resource scheduling decisions.
- **Hardware Setup:** Experiments are run on Google Cloud Platform (n1-standard-16) VMs with 16 vCPUs and 60 GB RAM. Results are averaged over five trials with distinct random seeds to ensure statistical validity.

The source code and configuration scripts will be made publicly available upon acceptance to facilitate reproducibility and future research.

### Training strategy and reproducibility

To ensure full reproducibility and transparency, we present the training configurations of all major components in Table 11, Table 12, and Table 13.

**Table 11:** Training Configuration for BiLSTM Forecaster.

Parameter	Value
Model Architecture	2-layer BiLSTM
Hidden Units per Layer	64
Attention Mechanism	Temporal Softmax
Loss Function	Pinball Loss ($\tau \in \{0.1, 0.5, 0.9\}$)
Optimizer	Adam ($\text {lr}=0.001$, $\beta _1=0.9$, $\beta _2=0.999$, $\epsilon =10^{-8}$)
Learning Scheduler	Cosine annealing with 5-epoch warm-up
Regularization	Dropout (rate = 0.3)
Batch Size	256
Max Epochs	100
Early Stopping	Patience = 10 (based on validation MAE)
Input Normalization	Z-score
Tuning Method	Bayesian Optimization (50 trials)

**Table 12:** Training Configuration for MARL Agents.

Parameter	Value
Policy Network	3 hidden layers (128, 128, 64), ReLU
Action Space	Continuous $[-1, 1]^3$ (scale, migrate, suspend)
Reward Weights	$\alpha =0.5$, $\beta =0.3$, $\gamma =0.2$
Optimizer	Adam (initial lr = $5 \times 10^{-4}$, linear decay)
Discount Factor	$\gamma = 0.99$
Exploration Strategy	Ornstein–Uhlenbeck ($\theta =0.15$, $\sigma =0.2$)
Replay Buffer Size	1 million transitions
Batch Size	512
Credit Assignment	Variance-Regularized

**Table 13:** Training Configuration for Ape-X Learners.

Parameter	Value
Number of Actors	32 parallel agents
Number of Learners	8 (GPU-distributed)
Priority Sampling	$p_i = |\delta _i|^{0.6} + 0.3 \cdot \sigma _{\text {BiLSTM}}(s_i)$
Target Update	Soft update with $\tau =0.005$
Sample Interval	Every 4 environment steps
Gradient Clipping	Max norm = 10

**Reproducibility Measures**

- 70/15/15 train/validation/test split maintained across all experiments
- Results averaged over 5 different random seeds
- Implemented in PyTorch, TensorFlow, and Ray RLlib (custom Ape-X)
- Hardware: Google Cloud (n1-standard-16 VMs), Tesla V100 GPUs

### Evaluation methodology

We adopt a rigorous evaluation strategy to ensure robust and generalizable conclusions.

- **Data Splitting:** A 70/15/15 train/validation/test split is used to evaluate the learning, tuning, and generalization phases.
- **Stress Testing:** A 24-hour stress test is conducted to simulate high-load, real-world scenarios and assess the resilience of the system.
- **Deployment Environment:** Experiments are deployed on Google Cloud Platform GCP instances to mimic real-world infrastructure setups.
- **Cost Analysis:** An economic evaluation is performed using the AWS EC2 pricing to analyze cost-effectiveness.

## Data Availability

The datasets used to evaluate the proposed framework are publicly available and can be accessed as follows: $\bullet$ **Google Cluster Trace**: Available at https://github.com/google/cluster-data. This dataset contains resource usage traces from Google’s production clusters, including CPU, memory, and disk usage over time. $\bullet$ **Azure Public Dataset**: Available at https://github.com/Azure/AzurePublicDataset. This dataset includes VM workload traces from Microsoft Azure, capturing resource utilization metrics such as CPU, memory, and network I/O. $\bullet$ **Bitbrains Trace**: Available at https://github.com/bitbrains. This dataset contains performance metrics from enterprise-level cloud workloads, including CPU utilization, memory usage, and disk I/O. These datasets were preprocessed and normalized for use in our experiments. The preprocessing scripts and detailed instructions for reproducing the results are available in our GitHub repository https://github.com/fadynashat/LSTMMARLAPe-x_Sol/.
